# High-resolution ultrasound of peripheral neuropathies in rheumatological patients: An overview of clinical applications and imaging findings

**DOI:** 10.3389/fmed.2022.984379

**Published:** 2022-10-31

**Authors:** Federico Zaottini, Riccardo Picasso, Federico Pistoia, Sara Sanguinetti, Michelle Pansecchi, Luca Tovt, Umberto Viglino, Corrado Cabona, Martina Garnero, Luana Benedetti, Carlo Martinoli

**Affiliations:** ^1^San Martino Hospital, Istituto di Ricovero e Cura a Carattere Scientifico, Genoa, Italy; ^2^Dipartimento di Medicina Sperimentale, Scuola di Scienze Mediche e Farmaceutiche, Università di Genova, Genoa, Italy; ^3^Dipartimento di Scienze della Salute, Scuola di Scienze Mediche e Farmaceutiche, Università di Genova, Genoa, Italy; ^4^Eye Clinic, Department of Neuroscience, Rehabilitation, Ophthalmology, Genetics, Maternal and Child Science, School of Medical and Pharmaceutical Sciences, University of Genoa, Genoa, Italy

**Keywords:** ultrasound, nerve imaging, polyneuropathy etiology, rheumatologic conditions, magnetic resonance imaging

## Abstract

Peripheral neuropathies are surprisingly common and can be associated with a number of conditions, including rheumatological diseases. Whether the co-existence of peripheral neuropathies with rheumatological disorders is coincidental or related to a common pathogenic mechanism, these disabling conditions can affect the outcome of rheumatological patients and should be targeted with specific treatment. The clinical presentation of peripheral neuropathy can be multifaceted and difficult to recognize in polysymptomatic patients. However, physicians adopting state-of-art diagnostic strategies, including nerve imaging, may improve the detection rate and management of neuropathies. In particular, a diagnostic approach relying exclusively on clinical history and nerve conduction studies may not be sufficient to disclose the etiology of the nerve damage and its anatomical location and thus requires integration with morphological studies. High-Resolution Ultrasound (HRUS) is increasingly adopted to support the diagnosis and follow-up of both joint disorders in rheumatology and peripheral neuropathies of different etiologies. In this review, the different types of nerve disorders associated with the most common syndromes of rheumatological interest are discussed, focusing on the distinctive sonographic features.

## Introduction

Peripheral neuropathies (PNs) are frequently encountered in the context of rheumatic disease (1). However, the pathogenic mechanisms determining this association are multiple and partially still unrevealed. Indeed, PN in musculoskeletal inflammatory disorders can be directly caused by a wide variety of mechanisms, such as compression, vasa nervorum inflammation, or drug toxicity. In other circumstances, rheumatic disease and coexisting neuropathy share an immune-mediated mechanism. Finally, in some instances, the link between the two conditions cannot be firmly proven, as is the case of small-fiber neuropathies.

In the last years, we have witnessed an increasing use of ultrasound (US) for assessing patients with inflammatory arthritis. In addition to being an inexpensive, safe and widely available tool, the use of US allows a more accurate assessment of the soft tissue inflammation compared to clinical examination also providing the same sensitivity as magnetic resonance imaging (MRI) (2, 3).

Furthermore, the recent advances in electronics and signal filtering algorithms, together with the innovation of high-frequency broadband linear transducers (>18 MHz), have all contributed to increasing the spatial resolution of US and its diagnostic performance. This technology known as High-Resolution Ultrasound (HRUS) is excellent for rapidly assessing long nerve tracts in the extremities (4). Moreover, this technical upgrade has allowed US to become an increasingly useful diagnostic tool for the differentiation of the pathogenetic mechanisms underlying a particular neuropathy, such as entrapment neuropathy, traumatic neuropathy, or inflammatory polyneuropathies (5–9). In addition, HRUS is non-invasive and is an easily available diagnostic instrument that can provide promising imaging biomarkers (10) for a neuropathy follow-up either after treatment and/or during rehabilitation protocol.

Since rheumatic patients frequently seek medical advice for symptoms that are not always attributable to joint involvement or to extra-articular causes, a thorough knowledge of US findings, characterizing the different subtypes of neuropathies, may well help in promptly recognizing the subgroup of rheumatic patients affected by neuropathies, thus improving the appropriate clinical management of their conditions. The purpose of this narrative review is to provide an overview of the clinical and HRUS features of the neuropathies associated with rheumatic diseases, summarizing the literature evidences regarding these conditions.

## Classification of neuropathies and clinical diagnosis

Peripheral neuropathies can be categorized in different types according to specific criteria (11). The most commonly applied criterion used today by neuropathologists is based on the anatomical structure involved. Therefore, these conditions are classified as Axonopathy, Myelinopathy, Ganglionopathy, or Neuronopathy each according to the neural structure involved. Considering the number of nerves affected, it is said to be mono-neuropathy if a single nerve is damaged, multifocal neuropathy if at least two separate nerve areas are asymmetrically and asynchronously involved, or polyneuropathy when multiple nerves are affected symmetrically. In addition, according to the type of function, neuropathies can also be divided into sensory, motor, autonomic, or mixed neuropathies, while based on the subtypes of fibers affected, they can be classified as large fiber or small fiber neuropathies. Finally, these conditions can be further divided into acute or chronic neuropathies depending on their onset and progression. The whole spectrum of these neuropathies can be found in rheumatological patients.

In a clinical setting, the different subtypes of neuropathies are defined according to the nerve conduction studies (NCS), which typically correspond to specific symptoms.

Single mono-neuropathies are characterized by sensory disturbances and a loss of muscle strength in the innervation territory of the affected nerve, as commonly found in the case of compression neuropathies. In compression neuropathies, symptoms can range from sensory abnormalities, paresthesia, and pain in the initial stages to motor deficit and permanent sensory impairment as the injury progresses. Demyelination accounts for the slowing of the nerve conduction in the initial phase of the alteration with the prevalent involvement of sensitive fibers; as the damage progresses, motor fibers are deranged and the alteration reaches the axons (12).

**FIGURE 1:**
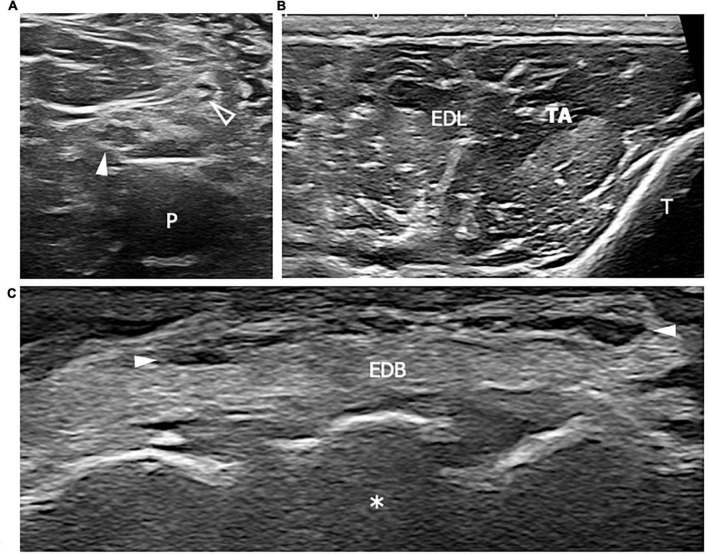
This figure shows three 18 MHz HRUS images **(A–C)** of a 44 years old male affected by a mixed connective tissue disease with a lower limb sensory-motor axonal neuropathy. As frequently seen in the axonal neuropathies, in **(A)** no significant morphological changes of the tibial (white arrowhead) and fibular (empty arrowhead) nerves in the popliteal fossa are seen. At the proximal third of the leg, the axial scan in **(B)** does not reveal any sign of denervation in the anterior compartment of the leg muscles (tibialis anterior, TA and extensor digitorum longus, EDL). However, the extensor digitorum brevis muscle (EDM) in **(C)** exhibits signs of advanced atrophy with fat replacement of the muscle fibers. The isolated atrophy of EDM has been described as being associated with axonal neuropathies of different etiologies and it could represent the only HRUS finding under these conditions. White arrowheads in **(C)** represents EDL tendons. P, popliteal vein; T, tibia; white asterisk, tarsal bones.

Occasionally, compression neuropathies may initiate with prevalent motor or sensory symptoms according to which group of fascicles is pre-eminently affected (13). Auto-immune demyelinating polyneuropathies are characterized by the symmetric or asymmetric slowing of conduction, more or less associated with conduction blocks that involve both motor and sensory fibers. This group of neuropathies, also include the Motor Multifocal Neuropathy (MMN), a purely motor neuropathy characterized by asymmetrical involvement, conduction blocks, and intact sensory nerve action potential. Axonal sensory polyneuropathy can be characterized by paresthesia and a sensory loss (including mild touch, proprioception, and vibration sensations) in the distal part of the limbs, mainly the lower limbs, and the affected patients may also complain of a burning, painful sensation in the feet. In addition to the sensory manifestations, motor weakness may be found in sensorimotor axonal polyneuropathy, usually affecting the extensor muscles of the toes or feet (11) (Figure 1). Selective motor neuropathies are characterized by paresis, atrophy and fasciculations, mainly in the distal limbs (11). Small-fiber neuropathy (SFN) results from selective damage to small myelinated and unmyelinated nerve fibers. SFN is usually found in the early stages of several systemic diseases, such as diabetes, amyloidosis and rheumatic conditions (14). The main clinical manifestations are numbness, a burning sensation, electric pain, a pricking sensation, and pruritus involving the limbs, trunk or face (11). SFN consists of two different types, the “length-dependent” SFN and the “non-length-dependent” SFN. The first one is related to the most distal axon degenerations causing neuropathic pain arising in a distal “stocking-and-glove” distribution; in the second type, the neuronal degeneration involves the proximal region of the peripheral nervous system and dorsal root ganglia; these patients suffer from neuropathic pain in the face, truncus, and proximal arms and legs (15). However, it is worth noting that NCS cannot distinguish between small fibers or amyelinic fibers pathology and a conventional electromyogram or NCS are non-contributory to the diagnosis of a small fiber neuropathy (16).

## High-resolution ultrasound findings

As a consequence of the variable clinical manifestations, the diagnosis of PN is a challenging clinical issue, moreover in polysymptomatic disorders such as rheumatic diseases. HRUS may represent the first screening instrument to discriminate nerve damage from joint and soft tissue involvement. In addition, the morphological information provided by HRUS complements the NCS and clinical information to unveil the etiology of PN, the potential site of injury and the extent of nerve alterations (17). The different types of PNs found in rheumatological conditions may occur by way of several mechanisms, including the direct effects of compression or the invasion of neural tissues by hypertrophic synovium, vasculitic involvement of the vasa nervorum, auto-immune mechanisms directed against the myelin antigens, occasionally triggered by biological drugs, or the direct neurotoxic effect of drugs or deposited material (e.g., amyloids).

### Scanning technique and normal nerve appearance

Ultrasound study of the nerves should be conducted according the so called “elevator technique” (18). It consists in using anatomic landmarks to find the target nerve in its short axis and scanning it cranially and caudally along the entire course. In short axis, normal peripheral nerves demonstrates a characteristic honeycomb-like appearance, related to the presence of hypoechoic axons arranged in fascicles and multiple layers of hyperechoic connective tissue surrounding the axons bundles. In the long axis, the nerve appears as elongated structures with alternating hypo-and hyperechoic bands. The ultrahigh frequency probes (up to 30 MHz) allow visualization of sub-millimetric terminal nerve branches, which are visualized like a single or few hypoechoic dots within a hyperechoic frame lacking the classical expected “honeycomb-like” structure (18). Pathological changes may be focal or diffuse, and are typically recognized at ultrasound as nerve enlargement respect to normal ones and/or changes in the fascicular echotexture. In order to avoid misdiagnosis, the probe should be kept exactly perpendicular to the nerve long axis, otherwise size measurements and echogenicity evaluation may be altered respectively by the obliquity of nerve cross section and the anisotropy. Anisotropy is an artifact typically encountered during scanning of fibrillar structures as muscles and tendons, but also nerves. In fact, when the ultrasound beam is incident on organized fibrils or fascicles, may be reflected in a direction away from the transducer: in those cases, the transducer does not receive the returning echo and the insonated area is displayed hypoechoic. Restoration of the beam perpendicular angle of incidence by tilting the probe, promptly resolve the false image (19). Keeping into consideration the potentials diagnosis pitfalls ensuing an inadequate scanning technique, in the next subparagraphs the sonographic findings of neuropathies are described in relation to the pathogenesis.

### Compression neuropathies

**FIGURE 2:**
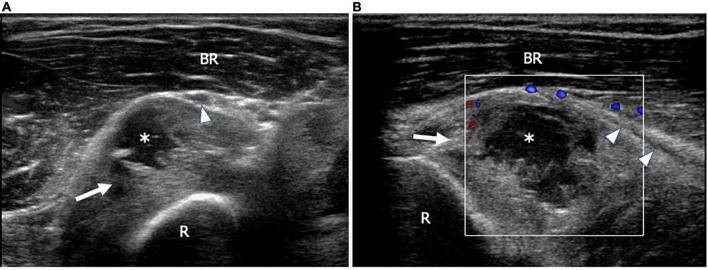
This figure shows two 18 MHz HRUS images **(A,B)** of a 53 years old male patient affected by rheumatoid arthritis with a progressive decrease of fingers extension strength. **(A)** An axial view, immediately distal to the humeral-radial joint line, shows the annular recess (white arrow) distended by synovium and fluid (white asterisk); the recess dislocates superficially the posterior interosseus nerve (white arrowhead) immediately proximal to the supinator tunnel. After switching on the Eco-Color Doppler, a slight peripheral vascularization of the synovial wall of the annular recess (white arrow) is shown **(B)**. **(B)** The longitudinal scan also depicts the long axis of the posterior interosseous nerve (white arrowheads) dislocated superficially by the distended recess. BR, brachioradialis muscle; R, radius.

Compression mono-neuropathies are relatively frequent conditions in rheumatological diseases with synovial hypertrophy or with structural changes in the connective tissue [i.e., rheumatoid arthritis (RA) or systemic sclerosis] which may, directly or indirectly, create a mass effect on the nerve, especially within the osteofibrous tunnels (Figure 2). Regardless of the entrapment site, HRUS signs of compressive neuropathies are stereotypical and consist of nerve flattening at the compression point and nerve swelling proximally or (less commonly) distally to it. In case the transition between swollen and flattened segments is abruct, it represents the patognomonic “notch sign.” In order to have the quantification of findings, the nerve cross-sectional area (CSA) or the maximum nerve diameter should be sampled at the site where the nerve is maximally enlarged proximally or distally the compression site, and this value can be used to differentiate normal from compressed nerve (20). In particular, the measured CSA can be compared to a validated cut-off, to the contralateral nerve or to a normal tract from the examined nerve. According the EFSUMB (European Federation of Societies for Ultrasound in Medicine and Biology) guidelines, for the Ultrasound diagnosis of carpal tunnel syndrome (CTS), the CSA threshold value of 10 mm^2^ obtained proximal to the flexor retinaculum should be used to differentiate a normal from compressed median nerve. Similarly, an ulnar nerve CSA cut-off value of 10 mm^2^ obtained proximal or within retrocondylar groove at the elbow, should be considered for the diagnosis of cubital tunnel syndrome (21).

Nerve CSA is seen to correlate with NCS severity grading, since nerve enlargement corresponds to pathologic changes that directly hamper the nerve functions. In the early phases of compression, intraneural edema and venous congestion are the main factors leading to an enlargement of the nerve and consequent myelin sheets alterations. In severe/long-standing compressions, the nerve loses the fascicular echotexture as a consequence of the swelling of the fascicles and progressive intraneural fibrosis. Differently from the early stages of the disease, nerves with fibrotic changes remain enlarged after decompressive surgery and have poor functional improvement (22, 23).

### Dysimmune neuropathies

In regards to auto-immunity, a link has been established between Chronic Inflammatory Demyelinating Polyneuropathy (CIDP) and other auto-immune diseases (24). In fact, certain HLA loci are associated with a greater risk of CIDP and other conditions, such as RA, systemic lupus erythematosus (SLE), and Sjögren’s syndrome (SS) (25, 26).

**FIGURE 3:**
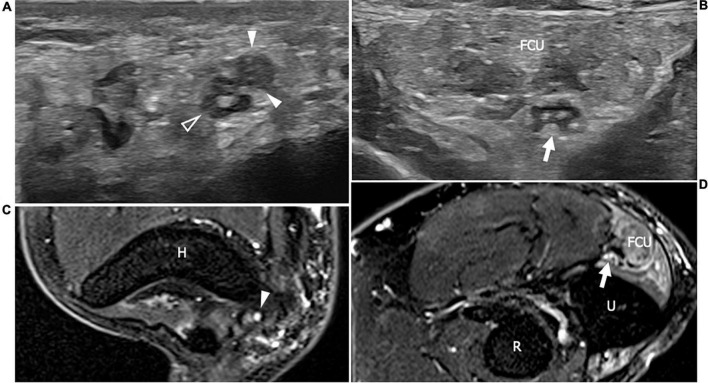
This figure shows two 18 MHz HRUS axial images **(A,B)** and two T2-weighted with fat suppression axial magnetic resonance (MR) images **(C,D)** in a 48 years old female patients with ANCA-associate vasculitis and a multifocal mononeuropathy of the ulnar nerve. **(A)** The axial view of the ulnar nerve (empty arrowhead) just cranial to the epitrochlear groove can be observed: the nerve is characterized by a single fascicle enlargement (white arrowheads) to which a focal T2-signal hyperintensity in the correlative MR image corresponds (white arrowhead in **C**). More distally, within the cubital tunnel, the ulnare nerve fascicles (white arrow) appear slightly enlarged and hypoechoic **(B)** with a s increase of T2-signal intensity in the matching axial MR image (white arrow in **D**). The flexor carpi ulnaris muscle (FCU) appears diffusely hyperechogenic in **(B)** as a consequence of denervation; in **(D)**, the denervation process provokes an intramuscular edema seen to have increased the T2-signal within the muscular tissue. H, humerus; R, radius; U, ulna.

**FIGURE 4:**
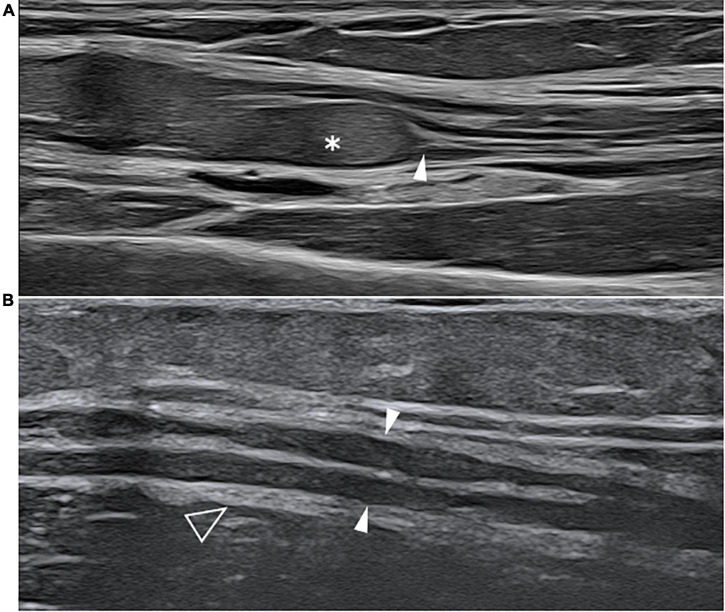
This figure shows two 22 MHz HRUS images, longitudinal view, one from a 45 years old female patient affected by systemic lupus erythematosus (SLE) who developed a chronic demyelinating polyneuropathy **(A)** and one from a 53 years old female patient affected by polyarteritis nodosa with a mononeuritis multiplex. The comparison of the two HRUS images point out the main distinguishing features of the two neuropathies: in **(A)**, there is a marked, iso-hyperechoic, irregular median nerve enlargement (white asterisk) with an abrupt change in caliber (white arrowhead) at the passage between the pathological and unaffected fascicles while in **(B)**, the ulnar nerve (white arrowheads) presents a slight, diffuse, irregular and hypoechogenic enlargement of the fascicle with a tick epineurium (empty arrowhead). Panel **(A)** was obtained at the mid-portion of the forearm while panel **(B)** was obtained at the distal third of the arm.

Furthermore, the introduction of immuno-modulating drugs, for example the tumor necrosis factor-α (TNF-α) inhibitors, has been associated with the development of auto-antibodies and immunity activation against peripheral nerve structures. The diagnostic value of HRUS has already been proven in acquired immune-mediated demyelinating neuropathies, either acute or chronic forms (27). Diffusely increased CSAs of the affected nerves, especially in the proximal extremities, and the focal fascicular enlargement at the site of conduction blocks are the sonographic hallmarks in demyelinating neuropathies. Fascicles may alternate in thickened and thinned segments and frequently results iso-hyperechogenic in comparison to the normal nerves as consequence of intraneural edema. The distribution pattern of nerve alterations can be diffuse, generalized, or focal according to the subtypes of demyelinating neuropathies (28). Nerve enlargement in dysimmune neuropathies in some cases is readly identifiable with a standard visual approach, mostly in the phase of active disease and in multifocal forms; in other cases, mainly in long standings, diffuse disease, nerve and fascicle swelling are mild, and a comparison of CSA measures with a matched control group or literature data is necessary to identify the pathological changes. In case of symmetric polyneuropathies as CIDP, the contralateral side comparison should not be used as diagnostic criteria for detecting nerve alterations. Recently introduced parameters as intranerve-, internerve-, and intra-plexus CSA variabilities and “side to side difference ratio of the intranerve CSA variability” can help into recognizing a diffuse, symmetric nerve involvement respect to multifocal or focal pattern (28, 29). In order to calculate these ratios, a multilevel symmetric CSA sampling should be obtained: in diffuse nerve involvement the variability in multilevel cross sectional area is reduced respect to multifocal or focal nerve alterations. Unfortunately, cut-off values for these parameters has been defined only for the ulnar nerve and there is low agreement between different studies, thus encouraging each ultrasound lab to define its own reference values. Differently from demyelinating neuropathies, nerve morphology is rarely abnormal in axonal polyneuropathies (30, 31). Vasculitic neuropathies may be an exception in the context of these findings. In fact, according to Leupold et al. (32), pronounced changes in different nerves may be detected in vasculitis using HRUS even if it shows the electro-diagnostic features of axonal neuropathy. Differently from demyelinating neuropathies, in vasculitic neuropathy, the mean CSA value sampled at different nerve levels bilaterally are normal or just slightly increased but focal nerve swelling, with impressively enlarged fascicles in the affected nerves, may be visualized (33). The enlarged fascicles are the results of ischemic axonal damage with Wallerian degeneration and the pattern of nerve involvement is typically multifocal (also known as “mononeuritis multiplex”). Other minor findings of vasculitic neuropathies include a decreased echogenicity of the fascicles with a hyperechogenic, thickened epineurium, which can persist after immunosuppression as described in a case report (34) (Figures 3, 4). Since the axonal neuropathies do not generally show a significant nerve enlargement at HRUS, nerve morphological alterations in the case of asymmetric axonal damage of unknown origin may suggest the diagnosis of nerve vasculitis.

Morphological and echotextural nerve alterations are detectable also in the acute form of autoimmune polyneuropathy, namely the Guillain-Barrè syndrome (GBS) and its variants (e.g., Miller Fisher syndrome). Among GBS subtypes, acute inflammatory demyelinating polyradiculoneuropathy and acute motor axonal neuropathy are the most common (35). According to HRUS findings in GBS, in the first three days of symptom onset, a diffuse nerve swelling involving the proximal segments (e.g., cervical roots) has been reported, although the frequency and extent of the morphological changes vary both inter- and intra-individually (35). The morphology of the purely sensory sural nerve was not significantly altered, which corresponds to the electrophysiological findings of “sural sparing” (36). The degree of cervical spinal nerve enlargement was found to be correlated significantly with the CSF protein and may represent the ultrasonic correlation of focal inflammatory demyelination with the swelling of the nerve sheaths, as reported in the histopathology (35). Nevertheless, the nerve’s echogenicity seems not to be altered in contrast to the descriptions in CIDP (35). The vagus nerve may also result morphologically altered and might well serve as an early risk marker of autonomic dysregulation. The proximal cervical nerve enlargement is reported to go back to normal 6 months after the beginning of symptoms and the start of therapy. However, the alterations in the distal nerve segments may last more than 6 months, possibly as a consequence of remyelination processes and/or Wallerian degeneration (36).

**FIGURE 5:**
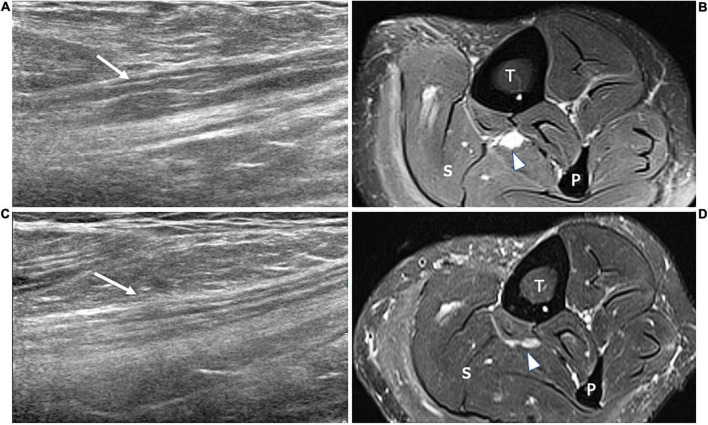
This figure includes two 18 MHz HRUS images **(A,C)** and two T2-weighted with fat suppression axial MR images **(B,D)** in a 45 years old female patient affected by rheumatoid arthritis who developed a motor multifocal neuropathy of the tibial nerve after starting a treatment with the anti-TNF-α drug Etanercept. **(A)** The longitudinal scan of the tibial nerve at the proximal leg shows the focal enlargement of the nerve fascicles (white arrow) to which corresponds the T2-signal hyperintensity (white arrowhead) of the MR image in **(B)**. Both the nerve size and the T2-signal alteration, compatible with intraneural edema ensuing an inflammatory process, result markedly reduced in **(B,D)**. These latter were acquired after 6 months of therapy with immunoglobulin. The findings of this figure highlight the role of HRUS in monitoring the disease activity of dysimmune demyelinating neuropathies. T, tibia; F, fibula; S, soleus muscle.

Within the setting of dysimmune neuropathies, HRUS is also able to demonstrate the response to systemic immunosuppressive treatment by showing a decrease in the nerve or in the fascicle cross-section area (Figure 5). Several study groups have found a correspondence between symptom variation and nerve morphological changes after therapy; in particular in chronic inflammatory demyelinating neuropathies. In addition, after immunoglobulin therapy a correlation between muscle strength and CSA of the affected nerves was demonstrated (37). Also in GBS, a regression of nerve enlargements might represent a good parameter for clinical recovery during follow-up (37, 38). Therefore, even though further studies are needed in order to prove the accuracy in demonstrating early signs of response to therapy or disease activity, nerve US might be a promising monitoring method.

### Muscle denervation

In the case of motor neuropathies, using HRUS, it is possible to identify and grade the ensuing muscle atrophy. A denervated muscle presents a variable appearance depending on the severity of axonal loss, durations of nerve damage and its evolution, the presence of reinnervation process. The muscle echogenicity is usually compared to subcutaneous tissue or the unaffected muscle, while the muscle volume is compared to the contralateral side. In literature are reported three pattern of echotextural alterations: no echo-intensity changes in case of monophasic, slight, axonal neuropathy; diffuse or patchy increased echogenicity in longer standing denervation without reinnervation; “moth eaten” pattern as consequence of chronic denervation and reinnervation processes. In this latter pattern, the hypoechoic areas correspond to viable, reinnervated motor units, that are interspersed in a hyperechoic background related to the fibrotic substitution of chronically denervated muscle bundles (39). Alterations in muscle echogenicity are indeed complex and may reflect denervation edema in the initial weeks after nerve injury followed by fat and fibrous tissue replacement of the muscle fibers with ongoing denervation (40).

Current literature is slightly contradicting on the ultrasound appearance of the muscle edema: according to some studies the edema determines muscle hyperechogenicity due to an increased number of reflecting interfaces (41). On the contrary, other studies state that edematous muscles appear swollen and hypoechogenic because of loosely packed peri and endomysial connective tissue (42). For this reason, in the acute phase (within the first 2-weeks) MRI is more sensitive and specific than ultrasound in detecting muscle denervation. As the fat replacement progresses, the muscle decrease in size and definitely increase in echogenicity (43).

In the recent years, software to quantify muscle thickness and echogenicity are more extensively adopted in clinical practice, in order to make a reproducible analysis of muscle denervation. By using quantification of muscle parameters, the severity of the ultrasound changes correlated with the severity of denervation on EMG. Furthermore, there is also potential for ultrasound measurements recorded from muscles to be included in disease assessment protocols (44).

In conclusion, HRUS may play a relevant role in the diagnosis and follow-up of neuropathies thanks to the number of anatomical details it can provide relating to the nerves and to the muscle, as well as its non-invasiveness and its availability for any care-setting environment. In the face of a spatial and contrast resolution not inferior to magnetic resonance neurography at least for the superficial nerves, further HRUS advantages include cost benefits, portability, dynamic imaging, and contra-lateral side comparisons. In addition, HRUS can also be performed on patients who are unable to tolerate MRI or patients carrying metallic hardware (45). However, among its limitations, we should mention the lack of a standardized methodology for US measurements, as well as the influence of the equipment, patients habitus and the nerve anatomy (whether superficial or deep, the presence of fibrosis in the overlying tissue, etc.) on US diagnostic performance. In fact, these factors, taken altogether, limit the repeatability and reproducibility of the studies. Recently, the operator dependency of US findings has been disproved by a study from Telleman et al. (46). However, for HRUS to be effective, a long learning curve of the operator is required (46).

## Neuropathies in the most common acquired rheumatological conditions and systemic conditions of rheumatological interest

In this paragraph, the epidemiology, clinical features, patho-mechanism and sonographic features of PNs in acquired rheumatologic conditions most commonly encountered in clinical practice are discussed (Table 1).

**TABLE 1:** This table shows the different types of neuropathies (first row) that may be found in each Rheumatological disease (first column).

	Acute demyelinating neuropathy (GBS-like)	Chronic demyelinating polyneuropathy (CIDPS-like)	Multifocal mononeuro pathy	Compressive neuropathy	Sensory-motor axonal neuropathy	Small-fibers neuropathy
Systemic Lupus Erythematosus	+	-	-	-	+++	-
Sjogren’s Syndrome	-	++	+++	+	++	-
Systemic Sclerosis	-	-	-	++	++	+
Rheumatoid Arthritis	-	-	+	++	+++	-
Polyarteritis Nodosa	-	-	+++	-	-	-
ANCA-Associated Vasculitis	+	-	+++	-	++	-
Giant Cell Arteritis	-	-	+	++	+	-
Takayasu Arteritis	-	-	-	+	+	-
Behcet Syndrome	-	-	-	-	++	-
Psoriatic Arthritis	-	-	-	+	+	+
Mixed Connective Tissue Disease	-	-	-	++	++	-
Sarcoydosis	-	+	+	+	++	+
Gout and Pseudogout	-	-	-	+++	+	+
Dermato-Polymyositis	-	-	-	-	++	-
Drug-related neuropathies	++	++	+	-	-	++
Covid-related neuropathies	++	++	+	+	+	-
Amyloidosis	-	-	+	+++	-	+

The relative frequency of a neuropathy in respect to the other types is indicated by the number of + (+++, frequently present; ++, present; +, rarely present).

### Systemic lupus erythematosus

Patients affected by Systemic Lupus Erytehamatosus (SLE) may suffer from PN in a percentage ranging from 1.5 to 36% (47–53). However, studies that considered only NCS parameter and not the clinical symptoms reported a prevalence of 21–42%, suggesting that some patients may present asymptomatic neuropathy (51). While NCS is a marker of peripheral nerve function, the significance of asymptomatic electrophysiological neuropathy is unclear. NCS parameters of SLE may deteriorate over time and the worsening seems related only to the age (48). According to some authors (54–56), SLE may cause an early damage to the peripheral nerve function before it can be detected by NCS or the patient experiences the initial symptoms: the clinical manifestations of PN are indeed the result of a slowly cumulative damage, as can be inferred by their correlation with age. Concerning the type of neuropathies, a higher prevalence of sensory-motor involvement with axonal patterns is reported (49). The most commonly affected nerves are in decreasing order the peroneal nerve, the tibial, and sural nerves, while the ulnar and median nerves involvement is uncommon (54). Although GBS is a rare type of PN in SLE patients, it represent one of the main causes of morbidity and mortality (57, 58).

The pathogenetic mechanisms of PN directly attributable to SLE neuropathy are not completely understood, but may include auto-antibody-mediated nerve damage and nerve vasculitis. And interestingly, the 30–40% of PN occurring in SLE are coincidental, related, for example, to diabetes mellitus, chronic renal failure, drug toxicity, or compressive neuropathy (59). The relation between PN occurrence and SLE disease activity or systemic involvement is not univocal: in some studies the symptomatic polyneuropathy was independently associated only with age while other case series have found associations with age onset and SLE disease activity scores (51, 59). Regarding nerve imaging, only one study has investigated the morphological features of PN related to SLE (60): in a cohort of 37 patients, the mean CSA of the ulnar nerve at the Guyon’s canal and mid-humerus, of the tibial nerve at the distal leg and proximal to the tarsal tunnel and of the peroneal nerve at the popliteal fossa resulted significantly increased in respect to gender and age-matched healthy control group. As already reported, HRUS is less informative in axonal neuropathies since the findings are less remarkable. Although the axonal polyneuropathies are the more represented subtypes among SLE-neuropathies, further data is needed in order to increase our knowledge about morphological HRUS findings in SLE, in particular in regards to nerve echotexture and fascicular size in the early asymptomatic phase when NCS and clinical examinations are normal.

### Sjögren’s syndrome

Sjögren’s syndrome (SS) is the second most common chronic auto-immune rheumatic disease, considering both primary Sjögren’s syndrome (pSS) and secondary Sjögren’s syndrome (sSS). The frequency, prevalence, and diagnostic criteria for peripheral nervous system involvement in SS have been the object of several studies as well as the underlying pathogenetic mechanisms. The reported prevalence of PN varies from 19 to 72% and may represent the first clinical manifestation (61–65). A cross-sectional study from China in 2018 reported a higher prevalence of PN in sSS than in pSS patients (31.1 vs. 19%) (61). Concerning the types of PN in SS, several studies found a higher prevalence of symmetric sensorimotor polyneuropathy (axonal and demyelinating type) and symmetric sensory polyneuropathy (66, 67); however, mononeuropathy (as entrapment neuropathy) or mononeuritis multiplex is also frequently encountered (61). Atypical presentations consist of pure motor neuropathies, hypertrophic neuropathies, and ganglionopathies (61, 68, 69). Regarding pathogenetic mechanisms, vasculitis seems to be strongly related to nerve damage, while the role of T-cell infiltrates and their cytotoxic effect is still unknown. Auto-antibodies, in particular the anti-muscarinic receptor 3 Ab, are implicated in the development of autonomic neuropathies (70).

**FIGURE 6:**
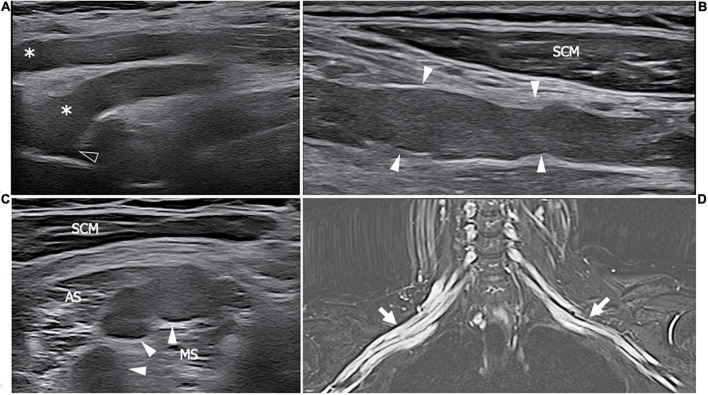
This figure shows three 18 MHz HRUS images **(A–C)** and a T2-weighted with fat-suppression magnetic resonance image **(D)** of a 50 years old female affected by SS who developed a predominant-upper limb sensory-motor demyelinating neuropathy. **(A)** A long-axis view of brachial plexus roots (white asterisk) at the exit from the intervertebral foramens (empty arrowhead) is obtained: the massive hypoechoic enlargement of the nerves with the complete loss of the typical fascicular echotexture is observed; these findings extend also more distally at the level brachial plexus trunks and divisions as seen in **(B)**. Also in the latter, the long axis view depicts the irregular profile of the nerves (white arrowheads), with areas of thinning and area of nerve enlargement, findings which are typical of a CIDP neuropathy. **(C)** A short axis view of the pathological nerve trunks (white arrows) in the interscalenic space is shown. **(D)** The MR coronal view of the neck and upper thorax demonstrates a massive, irregular enlargement of the brachial plexus bilaterally (white arrows); the increased T2-signal of the brachial plexus roots, trunks, divisions and cords is due to an intraneural edema consequential to the nerve inflammation. SCM, sternocleidomastoid muscle; AS, anterior scalene muscle; MS, middle scalene muscle.

Evidence about the imaging features of neurological involvement in SS is mainly focused on the central nervous system (CNS). The only ultrasound study found nerve morphological alterations in 72% of patients with suspected neuropathy related to SS. According to this study, nerve thickening is detected more frequently than fascicle thickening (90 vs. 52%, respectively), while in 40% of those patients combined nerve and fascicle thickening can be found (71). As observed in other autoimmune neuropathies, US abnormalities in the SS-associated neuropathy may occur in a single nerve site, as well as multifocally (71, 72). The morphological alterations reported in the study are consistent with the NCS diagnosis of demyelinating neuropathies, thus suggesting that this subtype of polyneuropathies is the most represented at least in the cohort examined by the authors (Figure 6). Further imaging studies based on HRUS are warranted on a larger cohort of subjects to disclose any nerve alterations in asymptomatic patients and to verify the utility of US nerve measurements as biomarkers of disease activity.

### Systemic sclerosis or scleroderma

**FIGURE 7:**
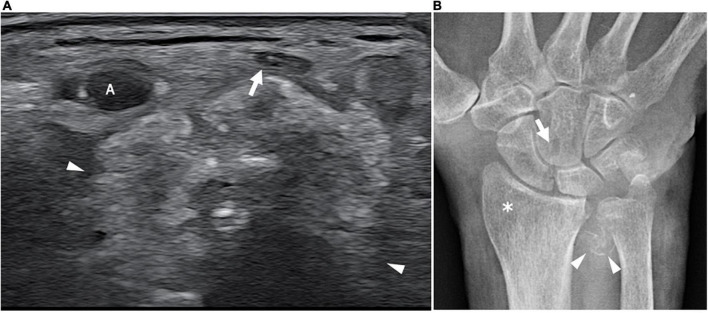
This figure shows an axial 18 MHz HRUS image of the wrist **(A)** and an antero-posterior X-ray image of the wrist in a 68 years old female affected by systemic sclerosis and monolateral carpal tunnel syndrome. **(A)** The distal radio-ulnar joint recess is distended by hyperechoic amorphous material partially calcified (white arrowheads) which compresses and superficially dislocates the median nerve (white arrow). **(B)** The X-ray confirms the presence of calcification (white arrowheads) in correspondence to the soft tissue in proximity to the distal radio-ulnar joint. Para-articular osteoporosis (white asterisk) and erosions (white arrow) at the level of the carpal bones are also observed. A, radial artery.

Systemic sclerosis (SSc) is an autoimmune disease characterized by vasculopathy, fibrosis, and immune abnormalities affecting the muscles, joints, skin, lungs, heart, digestive system, kidneys, and nervous system. Both central and peripheral nervous systems may be involved. In regard to PNs, in a cross-sectional study by Raja et al. (73) and Paik et al. (74), the prevalence of PN in SSc varied from 28 to 36.6%. In a recent systematic review from AlMehmadi et al. (75), compression neuropathies were reported in 26.5% of the studies, and according to Yagci et al. (76) and Sriwong et al. (77), the prevalence of median neuropathy in SSc is estimated to be 35%. Most CTS in patients with SSc were asymptomatic. Among non-compressive neuropathies, sensorimotor neuropathies (usually presenting as mononeuropathies) are the most frequent, whilst small-fiber neuropathies are detected more frequently than large-fiber neuropathies. Several etiologies were proposed, including intra-nerve fibrosis secondary to tissue edema. Calcinosis cutis and, less commonly, soft tissue thickening, resulted in the main risk factor for compression neuropathies in SSc (Figure 7). Risk factors for non-compression neuropathies include advanced diffuse diseases, anti-centromere antibodies, presence of vasculitis, iron deficiency with anemia, drugs such as metoclopramide and pembrolizumab, silicosis, and uremia (77). Autonomic nervous systems, especially cardiac autonomic functions, may also be altered in SSc (78). Literature evidence on nerve sonography in SSc patients is limited to the compression neuropathies. Tagliafico et al. systematically studied the median and ulnar nerves along their entire course in the upper limbs to find, as pathological alterations, nerve enlargement just proximal to the osteofibrous tunnel (the carpal tunnel and cubital tunnel), compatible with compression neuropathies. Occasionally, calcific deposits were found around the nerve (79). However, no sign of dysimmune neuropathies or vasculitis neuropathies were detected. Interestingly, Yagci et al. performed an elastography study on the median nerve revealing that the nerve loses its elasticity while the CSA’s is in the normal range in patients with SSC. The increased intraneural stiffness is likely to be related to the intraneural fibrosis, a finding which is also supported by the decreased “nerve density” evaluated using HRUS reported by Bignotti et al. (80). In fact, the computer-aided sonographic quantitative assessment of intraneural hyperechoic pixel/hypoechoic pixel ratio, demonstrated an increase of hyperechoic tissue in the nerves of patients affected by cutaneous Scleroderma, possibly related to an increased fibrotic tissue and/or decrease of fascicular size. The nerve density also resulted in being inferior in symptomatic patients with respect to the asymptomatic ones.

### Rheumatoid arthritis

The prevalence of PN in RA ranges from 10.81% (81) to 75.28% (82) according to the method of diagnosis, length of illness, and variability in selection criteria; the prevalence estimation is further complicated by the large fraction of asymptomatic patients. The chance of being affected by PN seems to vary according to the subtypes of RA considered. Kumar et al. (83) reported that the prevalence of PN in seropositive RA patients and seronegative RA patients was 34.4 vs. 15.4, respectively. However, the relationship between the presence of anti-cyclic citrullinated antibodies and the higher risk of developing PN needs further investigation. Among RA patients with the long-standing disease, sensory neuropathy is the most common form of PN (84), followed in order by distal sensorimotor polyneuropathy, mononeuritis multiplex, entrapment neuropathy, and neuropathy related to drug toxicity (84). Neuropathic abnormalities detected by NCS are most often axonal, although demyelinating features are sometimes present (85). Kaeley et al. (82) found that pure motor neuropathy was not a rare entity. The patient’s age, disease duration, use of disease-modifying anti-rheumatoid drugs, disease severity (disease activity score-28) and presence of subcutaneous nodules were found to correlate with the incidence of PN. Autonomic dysfunction also occurred in RA, characterized by heart rate responses to a deep breath, heart rate response to standing, blood pressure response to hand grip and sudomotor function impairment (86). Uncommon but severe peripheral nerve alterations included ischemic neuropathies caused by necrotizing arteritis of the vasa vasorum (87), presenting as multifocal nerve involvement. Entrapment neuropathy in RA is frequently related to synovial proliferation and joint deformation thus leading to nerve compression in proximity to the osteofibrous tunnel (88). No correlation was found between compression neuropathies and sex, duration of RA, functional class, the occurrence of other extra-articular manifestations, seropositivity, or the level of the acute phase plasma proteins. CTS is the most frequent compressive neuropathy in RA, but other entrapment neuropathies are reported, such as anterior and posterior tarsal tunnel syndrome (involving respectively a branch of deep peroneal nerve and the tibial nerve), the posterior interosseous nerve entrapment within the supinator tunnel, the cubital tunnel sydrome (involving the ulnar nerve at the elbow) and the common peroneal nerve compression at the fibular neck (88–90). The posterior tarsal tunnel syndrome is due to the compression of the tibial nerve as it enters in the tarsal tunnel, an osteo-fibrous canal delimited by the flexor retinaculum and medial malleolus and containing the tibialis posterior, flexor digitorum longus, and flexor hallucis longus tendons (89). The compression may results from tenosynovitis, the inflammation of the flexor retinaculum, or the pes valgus deformity. Symptoms include paresthesia and pain on the plantar aspect of the foot and the first through to the third toes. If the nerve compression persists, in the later stages atrophy and weakness of the intrinsic foot muscles occur as consequence of motor denervation. Prior to the availability of biological medications, from 5 to 25% of RA patients had electrophysiological abnormalities which are characteristic of tarsal tunnel syndrome, although not all of these patients were symptomatic (89). Median nerve entrapment commonly occurs at the wrist, within the carpal tunnel, as consequence of flexor tendons tenosynovitis or distension of radiocarpal and midcarpal volar recesses. Rarely, the compression can be identified in a more cranial position, at the level of the pronator muscle (pronator teres syndrome). Similarly to the median, ulnar nerve entrapment may be found at the elbow, within the cubital tunnel determined by distension of ulno-humeral joint recesses or at the wrist, within the Guyon canal; in this latter osteo-fibrous space, the ulnar nerve run in close proximity to the piso-triquetral joint, whilst, more distally, outside the tunnel, the motor branch may be compressed by a ganglion cyst from the metacarpophalangeal joint. The hip joint recesses and psoas bursa may determine femoral nerve palsy leading to sensory and motor defects (90). The tibial nerve may be compressed by a popliteal cyst, while ganglion cyst from the proximal tibio-peroneal joint may involve the peroneal nerve and its division branches. In the case of peripheral nerve entrapment and a concurrent cervical roots compression, the resulting syndrome has been termed “double crush” syndrome; the exact prevalence of this syndrome is not reported, however, considering the frequency of cervical spine involvement, it should always considered among differentials diagnosis of PN in RA.

Sonographic imaging research mainly considered CTS in patients affected by RA, even if the whole spectrum of compression and part of non-compression neuropathies are amenable to HRUS.

The sonographic features of CTS in RA patients are defined by the presence of finger flexor tendons tenosynovitis and/or radio-carpal joint synovitis which lead to median nerve compression. Conversely, a marked median nerve swelling in absence of the “inflammatory pattern” above described is the dominant feature in idiopathic CTS (91), in which other factors such as the flexor retinaculum thickness play a more relevant role in the pathogenesis of nerve compression.

### Polyarteritis nodosa

Polyarteritis nodosa (PAN) is a systemic necrotizing vasculitis of the medium and small sized muscular arteries. Among vasculites, PAN presents one of the highest PN prevalence. In a study on 27 patients from India, the prevalence reported is 88.9% (22 of 27 cases) (92). According to de Boysson et al. (93) and Imboden et al. (94), between 65 and 85% of PAN patients suffer from peripheral nerve disorders. Mononeuritis multiplex with axonal-type alteration affecting the major nerve trunks is the main form of PN manifestation, probably because PAN mainly affects the medium-size vessels, which supply slightly larger nerves. However, polyneuropathies, radiculopathies, lumbar or brachial plexopathies have all been reported. The chronic forms are predominant, even if rare cases of acute neuropathy related to necrotizing vasculitis may occur (95). As above described in the dysimmune neuropathies section and also reported by Kara et al., multifocal, hypoechoic nerve enlargements are the main sonographic features of mononeuritis multiplex related to vasculitis (96). The pathophysiology of nerve enlargement and fascicular swelling in vasculitic neuropathies remains unclear. The inflammation of the vasa nervorum, mainly affecting the epineural arteries may represent the first step leading to ischemic damage of the axon and consequent focal edema (96).

### Anti-neutrophil cytoplasmic antibody-associated vasculitis

Anti-neutrophil cytoplasmic antibody (ANCA)-associated vasculitis is a systemic disorder that encompasses three distinct conditions: microscopic polyangiitis (MPA), granulomatosis with polyangiitis (GPA, previously Wegener’s granulomatosis), and eosinophilic granulomatosis with polyangiitis (EGPA, previously Churg-Strauss syndrome). In order to clarify the pathogenetic mechanism, Nishi et al. (97) compared sural nerve biopsy specimens of 27 EGPA patients with anti-myeloperoxidase-ANCA and 55 EGPA patients specimens without anti-myeloperoxidase-ANCA. They found that the positive anti-myeloperoxidase-ANCA group was mainly characterized by vasculitis in epineural vessels, while the negative anti-myeloperoxidase MPO-ANCA group was mainly characterized by eosinophil infiltration, suggesting the existence of at least two distinct mechanisms. In a large cross-sectional study from Bischof et al. (98), the prevalence of peripheral nerve involvement was 65% in EGPA, 23% in MPA, and 19% in GPA. The characteristics of neuropathies caused by small vessel vasculitis, as the ANCA-associated vasculitis, are similar (99). The symptoms develop acutely in a single nerve territory followed by the gradual involvement of other nerves. At the beginning of the disease, the sensory symptoms are limited to one or few body areas, reproducing the typical pattern of mononeuritis multiplex; as the damage progressively involves multiple nerves, the clinical manifestations may reproduce the pattern observed in polyneuropathies (100). Often sensory disturbances are associated with muscle weakness and atrophy, whereas pure motor neuropathy is an exclusion criterion for vasculitic neuropathy (101). Mononeuritis multiplex is reported in approximately 70–90% of patients, probably due to the immunosuppressive treatment which limits the nerve damage thus avoiding the progression to symmetric or asymmetric polyneuropathy. In EGPA, the nerves in the lower extremities are primarily affected, with the peroneal nerve being the most frequently and severely involved. Patients affected by ANCA- vasculitis may also have symptoms of autonomic dysfunction which is independent of the disease duration and its severity (102). Atypical manifestations include acute sciatic nerve neuropathy (103) and GBS-like syndrome (104).

Excluding the typical ultrasound findings of mononeuritis multiplex, enlargement of the peroneal nerve and sural nerves have been described in small vessel vasculitis, in particular, the peroneal nerve CSA came-back larger in the vasculitis patient group than in the non-immuno-mediated, idiopathic neuropathy group (105).

### Giant cell arteritis and Takayasu arteritis

Reported evidence regarding PN in the large vessels vasculitis such as giant cell arteritis (GCA) and Takayasu arteritis (TAK) are scant. A literature review by Bougea et al. (106) found that PN affected 15% of GCA patients. CTS is the most common neuropathic complication of GCA, while mononeuritis multiplex and distal symmetrical sensorimotor polyneuropathies result uncommon. A rare neurological complication is the bilateral acute brachial radiculo-plexopathy, presenting as diplegia of the upper extremities with intact mobility of the head and lower limbs (107, 108). Also some cases with atypical symptoms, such as compressive common peroneal neuropathy (109) have been reported. PNs in TAK are rarer (110), usually presenting as a subacute sensorimotor deficit in a cervicobrachial plexus distribution. Even if uncommon, compression neuropathies should be considered as possible causes of PN. In this regard, a case report by Kim et al. (111) described a brachial plexus compression injury caused by a right axillary artery aneurysm in a young female patient affected by TAK.

### Psoriatic arthritis

Peripheral nerve involvement in psoriatic arthritis (PA) has not been extensively indagated. However, according to the data from the DANBIO register (112), the presence of neuropathic pain is reported by 28% of Psoriatic patients assessed through the PainDETECT Questionnaire (PDQ), turning out to be more frequent respect to RA and seronegative Spondylarthritis. It is unclear whether the PDQ actually brings up the presence of peripheral neuropathic pain, or the central sensitization typical of fibromyalgia. In fact, PA neuropathy and fibromyalgia share a similar profile of sensory alterations, including pathological changes in small nerve fibers (113). Narayanaswami et al. (114) reported three cases of polyneuropathy associated with PA. The clinical and electrophysiologic features were consistent with a chronic, distal, symmetric sensorimotor axonal neuropathy. CTS is also frequently encountered: a clinical and sonographic study from Kaya Subaşı et al. (115) found CTS in 30% of RA patients and 41% in PA patients. In both conditions the frequency of CTS was higher with respect to the control group. The CTS in PSA is often associated with flexor tenosynovitis and radio-carpal joint synovitis. Summarizing, length-dependent SFN, symmetric axonal neuropathies, and CTS are the main causes of neuropathic pain in PA. However, further studies on a larger cohort of patients are needed to better characterize with NCS and HRUS the features and etiopathogenesis of PN in the autoimmune dermatoses.

### Behcet syndrome

Peripheral neuropathies involvement in Behcet syndrome (BD) is extremely rare. Few cases of distal symmetrical polyneuropathy and mononeuritis multiplex have been reported (116), whilst the overall prevalence of CTS reached 0.8% in a retrospective study. According to the functional and electrophysiological classification, the nerve alterations in BD are axonal and predominantly involve the lower extremities (116).

### Mixed connective tissue disease

The prevalence of PN is mixed connective tissue disease (MCTD) varies between 10 and 17% (117), including a relatively high percentage of trigeminal and bilateral facial nerve palsy (118). Vasculitic and compressive neuropathies such as CTS can also be observed (119), presenting the typical HRUS features described above.

### Dermatomyositis and polymyositis

According to previous studies, 7.5% of dermatomyositis (DM)/polymyositis (PM) patients suffer from polyneuropathy (120), mainly of the axonal type. Consequently, no specific HRUS pathological nerve findings have been reported and sonographic imaging should be used as a tool to rule out coincidental compressive or inflammatory neuropathies as the cause of muscle weakness and atrophy. Ultrasound may also help into differentiating a “myogenic pattern” of muscle alteration from a “neurogenic pattern” (121). IN DM and PM usually muscle alterations start with focal hyperechogenic areas which progressively extend to the entire muscle belly; furthermore, subcutaneous edema and calcifications or increased muscle vascularity at Eco-Color Doppler evaluation can be found. Conversely, in muscle denervation, a patchy to diffuse increase in muscle echogenicity with a myotomal pattern of involvement are observed (see section “Muscle denervation”).

### Sarcoidosis

In a recent narrative review from Tavee (122), peripheral nerve involvement in sarcoidosis (SA) is distinguished in granulomatous neuropathy (GN) and the more recently described non-granulomatous small fibers neuropathy (NGSFN). The two clinical entities differ for anatomo-pathologic features and clinical presentation. The GN occurs in 1% of SA patients and usually manifest as distal, symmetric or asymmetric axonal sensory motor polyneuropathy (123). Less commonly, CIDP pattern or motor multifocal pattern with electromyographic findings of demyelination or conduction blocks are reported (124). Neuropathic symptoms may present as the only clinical manifestation of SA, even if a subclinical systemic involvement commonly coexist. Differently, NGSFN is reported in the 32–44% of patients (125). Typically it starts after 2 years of SA systemic involvement as intermittent sensory disturbances in the distal extremities to become a continuous pain and paresthesia. The pathognomonic pathologic findings in GN are non-caseating granulomas in the perineurium, epineurium or endoneurium of the affected nerves. Rarely, soft tissue granulomas may infiltrates nerves causing acute or subacute mononeuropathies (126). However, granulomatous involvement cannot explain the generalized nerve damage of polyneuropathies: microvasculitis with or without vessels wall necrosis indeed may lead to ischemia and following axonal degeneration in the large myelinated fibers. The demyelination reported in some cases may represent the result of immune damage or be secondary to axonal atrophy. In NGSFN, the damage to the small myelinated A fibers and unmyelinated C-fibers, seems to be mediated by inflammatory cytokines.

Only one ultrasonographic study about nerve involvement in sarcoidosis has hitherto been reported (127). The authors found and increased cross sectional area of the ulnar, fibular, tibial, and sural nerve, associated with loss of fascicular echotexture or with preserved fascicular echotexture but increased size of nerve fascicles. The two types of findings are related to intraneural edema and axonal loss with Wallerian degeneration as consequence of ischemia (loss of fascicular echotexture) or to remodeling following demyelination/remyelination processes (increased size of the fascicles). Further studies are needed to confirm these findings, considering the small sample size (13 patients).

### Gout and pseudogout

Gout is one of the most prevalent inflammatory arthritis in the world. In a recent study on a large population (442 patients), prevalence of symptomatic PN was 11% (128); in another study on 162 patients, the prevalence of PN at the NCS was 65% (129). The main risk factor for PN in gout are disease duration, blood level of uric acid and the presence of tophi. The pathogenesis is mainly compressive, related to the presence of thophi along the nerve course, while the toxic effect of urate on axons is a hypothesis that still need to be confirmed with neuropathologic findings. The PNs most commonly encountered are compression mononeuropathies or sensorimotor axonal polyneuropathies. Literature evidences about nerve ultrasound in gout are mainly reports of compression neuropathies by urate crystals deposits in soft tissue, in proximity of osteo-fibrous tunnel. Median nerve compression due to gout tophi within the flexor tendon is described (130) as well as a case of foot drop caused by deposition of urate crystals along the course of fibular nerve at the knee (131).

Similarly to Gout, Calcium Pyrophosphate Dihydrate (CPPD) crystal deposition (pseudogout) may lead to compression neuropathies in the limbs. Several reports of median nerve neuropathy at the carpal tunnel or ulnar nerve disorders at the cubital tunnel studied with HRUS, described hyperechoic crystal deposition in the flexor tendons or in the posterior band of ulnar collateral ligament of the elbow, causing nerve compression (132–135). In conclusion, patients with gout or pseudogout and mononeuropathy symptoms, should be screened with HRUS for the presence of amorphous or punctate hyperechogenic material compressing or dislocating a nerve in proximity of a potential entrapment site.

### Drug-induced peripheral neuropathies

Drugs-related neuropathies should be considered among the differentials in rheumatological patients with peripheral nerve alterations, even though the link between PN and drug exposure is often limited to the temporal proximity without any other evidence. In recent years, the treatment of rheumatic conditions has seen a dramatic increase of new drugs which target specific molecules involved in immunity response. Biological immunomodulators, like adalimumab, infliximab, and etanercept, act as inhibitors of the TNF-α and are reported to cause different types of PNs with an incidence of 0.3–0.4 per 1,000 person-years (136, 137). This class of drugs can induce acute demyelinating neuropathy also known as GBS, the GBS variant Miller Fisher syndrome, chronic inflammatory demyelinating polyneuropathy, motor multifocal neuropathy, and axonal neuropathy (138). Nerve damage was attributed to the T-cell and humoral immune attack on the peripheral myelin, vasculitis-induced ischemia, and the inhibition of axon signaling (139). Janus kinase (JAK) inhibitors are included among the therapeutic options for RA, connective tissue diseases (140) and for hemato-oncological conditions. In a study of JAK1 and JAK2 inhibitor treatment of myelofibrosis, a new onset of PN was observed in 22% of patients who complained mainly of sensory symptoms (140). As most medication-induced neuropathies, this class of drugs affects the peripheral nerve axons and causes distal, symmetric, sensory-predominant axonal neuropathy with a progressive proximal extension. In fact, sensory myelinated (Aβ) fibers (i.e., those responsible for proprioception) or Dorsal Root Ganglia are the most sensitive to the toxins, whereas motor fibers, as well as thinly myelinated (Aδ) and unmyelinated ones (which convey temperature/pain and autonomic afferent fibers) seem more resistant to drug-induced damage (141). The immunosuppressive prodrug leflunomide is currently used as a disease-modifying treatment for RA. Also in association with this drug, axonal sensorimotor polyneuropathies are reported (142–145). The pathological analysis reported by Bharadwaj and Haroon based on three nerve biopsies from affected patients, demonstrated epineural perivascular inflammation altering the large and small myelinated nerve fibers, suggesting an axonopathy with features of vasculitis. However, other studies have reported non-specific axonal loss. The electrophysiological demonstration of neuropathy is delayed respect to the symptoms onset and is usually possible from 3 to 6 months after drug use.

Regarding ultrasound imaging of drug-induced neuropathies, to the best of our knowledge, no evidence exists regarding specific HRUS findings. Generally speaking, it is reasonable to expect that the demyelinating form of drug-related neuropathies shows the HRUS features already described in CIDP, whereas for axonal forms, US may result poorly informative. However, further imaging studies with electrodiagnostic and clinical correlation are needed to better characterize these conditions.

### COVID-19-related neuropathies

The progressively increasing number of COVID-19 patients and survivors worldwide is accompanied by a rising of neuromuscular and rheumatologic complications reports, likely related to both the virus itself but also to the treatment and hospitalization (146, 147). Arthralgia and myalgia are the most common musculoskeletal symptoms of COVID-19 that can be caused by acute arthritis or myositis of various etiology (viral, reactive, and crystal-arthritis) (148). Regarding peripheral nerves, one of the largest observational studies (214 patients) on the topic reported a peripheral nervous system involvement in up to 9% of COVID-19 patients (149). Different mechanisms have been suggested to cause nerve injury. The hypothetical ability of SARS-CoV-2 to invade peripheral nerves *via* the ACE2 receptor warrants further investigation (150, 151). Alternatively, the similarities between SARS-CoV-2 surface glycoproteins and glycoconjugates in human nervous tissue support the theory of “molecular mimicry” as the mechanism of injury. The COVID-19 vaccination too has been related to many cases of PN. In these cases, the immune-mediated activation against nerve antigens is likely caused by molecular mimicry mechanism and bystander activation, both of which may be triggered by the vaccination (152). The hypothesis of direct nerve insult from the vaccination is unlikely since neuropathies after the vaccination are often reported contra-lateral to the injection site (152).

**FIGURE 8:**
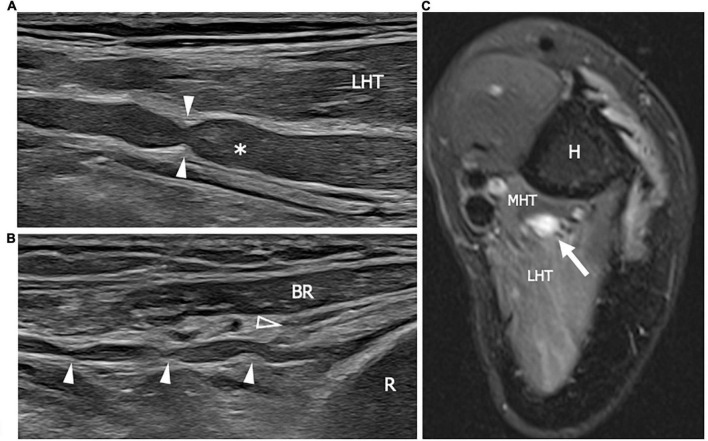
This figure shows two 22 MHz HRUS images **(A,B)** and one T2-weighted with fat saturation axial magnetic resonance image **(C)** in a 41 years old patient who developed a Parsonage-Turner syndrome following the vaccination against COVID-19. **(A)** The radial nerve (white asterisk) is seen in its long axis. The nerve results enlarged with a focal stricture (white arrowheads) along its course, deep to the lateral head of the triceps, proximal to the humerus spiral groove. The stricture is a sign of a torsion neuropathy, typically described in association with Parsonage-Turner syndrome. Multiple narrowing points (white arrowheads) can be observed also along the posterior interosseous nerve (PIN) course in **(B)**. The long axis view of the PIN shows the pathological alterations in the nerve tract proximal to the Frohse arcade or the supinator arch (empty arrowhead) which represents the likely fixation point predisposing to PIN torsion. **(C)** The radial nerve appears enlarged with an increase T2-signal as a consequence of intraneural edema. Notice the slight T2-signal hyperintensity of the lateral head and medial head of the triceps (LHT and MHT, respectively) related to muscle denervation. H, humerus; R, radius; BR, brachioradialis muscle.

The spectrum of neuropathies described following the COVID-19 infection or the COVID-19 vaccination include Parsonage-Turner syndrome, GBS, distal symmetric polyneuropathies, entrapment, and position-related neuropathies. High resolution US may help in differentiating the conditions since each of them is characterized by a specific pattern of nerve involvement, as described above. In particular the Parsonage-Turner syndrome may present distinguishing clinical and US findings. Parsonage-Turner syndrome or neuralgic amyotrophy, is an acute inflammatory-dysimmune mononeuropathy or multifocal mononeuropathy with a monophasic course, affecting mainly the brachial plexus and its branches (153). Four types of HRUS nerve abnormalities have been described: swelling, incomplete or complete focal constriction, and fascicular entwinement (154) (Figure 8). Although in specific conditions such as Parsonage-Turner syndrome, HRUS may be extremely informative and able to identify patognomomic findings, also for COVID-related musculoskeletal syndromes integration with MRI, clinical and electrodiagnostic studies is mandatory in order to reach the final diagnosis, and guide the therapeutic/rehabilitation plan.

### Amyloidosis

Amyloidosis includes a group of conditions with a common pathomechanism, consisting in extracellular deposition of insoluble, fibrillar proteinaceous material. In the affected patients, amyloid can be demonstrated in the joint and periarticular tissue, although the tissue distribution may vary considerably (155). The protein composition of the amyloid differentiates the amyloidosis types: serum A protein in the case of amyloidosis associated with chronic inflammatory diseases (aa amyloidosis), immunoglobulin light chain in AL amyloidosis, dialysis-associated β2-microglobulin in aβ2m amyloidosis, transthyretin (TTR) or other plasma protein in hereditary systemic amyloidosis.

Peripheral neuropathy is described in association with both hereditary and acquired amyloidosis. Sensorimotor axonal polyneuropathy, compression mononeuropathies such as CTS, or autonomic neuropathy (156) are the main PN in amyloidosis. Ultrasound findings in hereditary amyloid neuropathy have been the subject of several studies. Granata et al. found different morphological alterations associated with TTR-amyloidosis neuropathy which encompassed multiple areas of nerve enlargement as in multifocal neuropathy, bilateral enlargement in proximity of the osteofibrous tunnel (ulnar nerve at the cubital tunnel and median nerve at the carpal tunnel), bilateral nerve enlargement not related to the osteofibrous tunnel. However, the authors concluded that constant HRUS abnormalities were not present but findings differed on a case-by-case basis (156).

One of the most challenging differential diagnosis for amyloid polyneuropathy is CIDP. CIDP and amyloids may show significant overlap in NCS and clinical manifestation. HRUS may help to distinguish the two conditions by showing nerve fascicles uniformly enlarged in amyloidosis in contrast to CIDP, characterized by heterogeneous and irregular nerve swelling (157). Furthermore, TTR-patients have milder nerve enlargement with less variability in CSAs of median nerves than those with CIDP (158).

Finally, nerve HRUS may help in the early diagnosis and monitoring of pre-symptomatic TTR carriers. In fact, while in idiopathic CTS there is a direct correlation between clinical severity and median nerve CSA, in the subgroup of TTR subjects a dissociation between nerve morphology and electrodiagnostic findings is found. The morpho-functional dissociation can be considered typical of TTR-amyloidosis neuropathy. Furthermore, patients affected by symptomatic TTR-amyloidosis showed larger nerve CSA than pre-symptomatic carriers in several nerve sites, more pronounced at brachial plexus.

**FIGURE 9:**
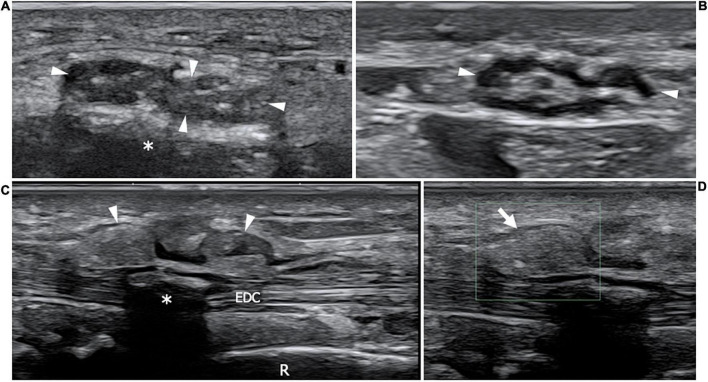
This figure shows two 22 MHz HRUS images **(A,B)** and two 18 MHz HRUS images **(C,D)** of a 62 years old male affected by multiple myeloma who developed an AL amyloid arthropathy. **(A)** An axial view of the median nerve (white arrowheads), situated in the proximity of the flexor retinaculum is presented. The nerve appears enlarged, with clumped hypoechoic fascicles, a finding which is related to the compression due to the underlying amorphous, hyperechoic material (white asterisk); the nerve echotextural alterations become even more evident if the image is compared to the image in **(B)** where the median nerve (white arrowheads) is visualized in the short axis at a more cranial level in the forearm. **(C)** On the dorsal aspect of the same wrist, a huge distension of the extensor digitorum communis tendons (EDC) synovial sheath (white arrowheads) can be observed. At the Eco-Color Doppler evaluation showed in **(D)**, the inhomogeneous, hyperechoic material distending the tenosynovial sheath (white arrow) results avascular, a finding which is compatible with an intrasynovial amyloid deposition. White asterisk, intra-tendon calcification; R, radius.

Differently from TTR-amyloidosis, little is known about nerve involvement in acquired amyloidosis. The majority of Ultrasound data about the musculoskeletal acquired amyloids has come from cases with musculoskeletal ß2 amyloidosis due to long-term dialysis (159). In a case report describing a patient affected by light-chain amyloidosis, the authors suggest a correlation between the entity of amyloid deposits and nerve enlargement seen with HRUS (160). The enlarged nerve is commonly accompanied by other sonographic findings in the surrounding soft tissue, notably tendon enlargement, synovial thickening and, synovia-like masses around tendons or within bursas (160). However, the absence of Doppler signal could help to distinguish these hypoechoic para-articular masses in respect to synovitis (Figure 9).

## Discussion

Although NCS along with clinical history and occasionally nerve biopsy constitute the well established diagnostic approach for the assessment of PNs in rheumatologic conditions, these tools present some shortcomings such as invasiveness and discomfort. In addition, clinical and electrodiagnostic approaches may produce false-negatives, show low positive predictive value and be non-localizing in patients with mild lesions or cases of severe axonal injury. By contrast, HRUS provides unparalleled complementary information which strengthens and accelerates PN diagnosis. Furthermore, HRUS is useful for the assessment of the response to treatment in inflammatory PNs. Even if the availability of 3T MRI with morphological and functional sequences is an appealing diagnostic tool, it should be considered as a complementary instrument to HRUS because it is costly, requires long scanning times and has lower spatial resolution than HRUS for the superficial nerves. However, PNs in rheumatologic diseases in most of the cases do not present specific sonographic patterns, but the US findings reproduce those reported in an idiopathic counterpart. Furthermore, at now, a significant percentage of neuropathies, mainly the axonal ones, are difficult to detect with HRUS. In view of the above, ultrasound is still extremely useful in differentiating a compressive neuropathies from a demyelinating autoimmune neuropathy or an amyloid neuropathy, all them potentially affecting the same rheumatologic patient but requires diverse therapeutic approaches. Our narrative review points out many potential triggers for further research regarding ultrasound applications in the diagnostic workup of the different neuropathies in rheumatology. In fact, in many conditions as LES, SS, the acquired forms of systemic amyloidosis, drug-related neuropathies etc., the scant imaging research focused mainly on entrapment neuropathies. However, the dysimmune polyneuropathies coexisting with autoimmune musculoskeletal disorders may be amenable to HRUS evaluation. In particular, it could be interesting to carry out morphological studies in those patients showing a mismatch between clinical neuropathic symptoms and NCS, frequently reported in many rheumatologic conditions: HRUS may result a useful, non-invasive screening tool for early recognition of nerve damage. Even those neuropathies which may result underdiagnosed with HRUS by applying a standard pattern recognition approach, could be better characterized by using quantitative parameters as nerve CSA, maximum and minimum fascicle diameter, intra-nerve and internerve CSA variability. Furthermore, the recent development of a radiomic type analysis seems to further increase the information that HRUS may yield. Radiomics is a relatively new approach for converting information and features present in medical images into quantifiable data. Moreover, machine learning algorithms have used these quantifiable data to enhance the diagnostic models. Ultrasound-based radiomics is a rapidly growing approach in imaging research that has already been applied to various organ (161). We expect in the next future the Ultrasound-based radiomics will be applied to nerves for a more precise identification of PNs.

## Author contributions

FZ and RP conceived and designed the review. FZ wrote the manuscript. FP, SS, MP, LT, UV, MG, CC, and LB collected the pathological cases and contributed to the literature search. CM supervised the study and contributed to the review design. All authors contributed to the article and approved the submitted version.
